# Benefits of targeted deployment of physician-led interprofessional pre-hospital teams on the care of critically ill and injured patients: a systematic review and meta-analysis

**DOI:** 10.1186/s13049-025-01347-w

**Published:** 2025-02-21

**Authors:** Adam J. Boulton, Terry Brown, Joyce Yeung

**Affiliations:** 1 Warwick Clinical Trials Unit, Warwick Medical School, University of Warwick, Coventry, UK; 2 Out-of-Hospital Cardiac Arrest Outcome (OHCAO) Project, Clinical Trials Unit, Warwick Medical School, University of Warwick, Coventry, UK; 3 Clinical Trials Unit, Warwick Medical School, Applied Research Collaboration West Midlands (ARCWM), University of Warwick, Coventry, UK; 4 Critical Care Unit, University Hospitals Birmingham NHS Foundation Trust, Birmingham, UK

Dear Editor,

We read with interest the systematic review and meta-analysis by Lavery et al. examining physician-led interprofessional pre-hospital teams for critically ill and injured patients [1]. As these teams continue to expand across international Emergency Medical Service systems, this study makes a timely and valuable contribution, reporting that physician-led interprofessional prehospital teams are associated with improved patient survival. The accompanying infographic and video have been effective in enhancing dissemination and engagement.

We have recently performed a similar systematic review and meta-analysis examining the impact of prehospital critical care teams in the care of out-of-hospital cardiac arrest (OHCA) patients [2]. Our findings are in keeping with Lavery et al., again identifying that these teams are associated with improved clinical outcomes compared to advanced life support care.

There are important differences between the reviews that we believe are worth highlighting and help develop the discussion surrounding physician-led teams in prehospital care. The population of interest is broad and does not focus solely on OHCA, whilst helpfully providing a subgroup analysis of this cohort. There is heterogeneity, with inclusion of trauma studies using different eligibility criteria (blunt trauma with reduced consciousness, blunt trauma with injury severity score (ISS) > 15, all patients with ISS 1–75, isolated severe traumatic brain injury etc.), as well as studies concerning OHCA. Similarly, there is heterogeneity in the comparator group, whereas our review focused on advanced life support care. By contrast, our review did not specify that prehospital critical care teams had to include physicians, however 16 of the 17 included studies had physicians present, highlighting the predominance of these physician-led teams in the published literature. Lavery et al. analysed studies reporting mortality and survival separately. Given the heterogeneity already present, we wonder whether combining these outcomes would have been beneficial to readers, noting the overall signal of benefit in both.

Both meta-analyses identify the benefit of these prehospital teams, with Lavery et al. expanding the reported advantages beyond OHCA [1, 2]. The eligibility criteria of Lavery et al. were broader. Twenty-three studies were captured, including 332,726 patients. Ten of these studies concerned OHCA, however our review identified 17 studies and included nearly 1.2 million patients, raising concerns that some relevant studies may have not been captured [3, 4]. There were differences in search timeframe, with Lavery et al. limiting to since 2010, however 15 of the studies included in our review were published since 2010. Our review also captured survival to hospital admission and favourable neurological outcome, finding improvements in outcomes. Whilst our review captured more OHCA studies and a wider set of clinical outcomes, the signal towards benefit is consistent across the reviews. Using the GRADE approach would have allowed ratings of the certainty of evidence. Our review found that the certainty of evidence for non-traumatic OHCA was low, and for paediatric and traumatic OHCA was very low [2]. 

Evidence from both reviews rely on non-randomised studies. Gaining higher quality evidence from randomised studies may be challenging due to concerns over equipoise, highlighting the importance of well-conducted observational studies in this area [5]. Neither review captures more nuanced outcomes, such as non-technical skills, team training and competencies, impact of individualised decision-making, including around termination of resuscitation [6]. Further pertinent questions remain, including which patients benefit most from these specialist teams, what enhanced interventions confer clinical benefit, how these teams can be expanded equitably, and what their cost-effectiveness is [7]. 

## Data Availability

No datasets were generated or analysed during the current study.
